# Olive Oil Consumption and Bone Microarchitecture in Spanish Women

**DOI:** 10.3390/nu10080968

**Published:** 2018-07-26

**Authors:** Raul Roncero-Martín, Ignacio Aliaga Vera, Luis J. Moreno-Corral, Jose M. Moran, Jesus M. Lavado-Garcia, Juan D. Pedrera-Zamorano, Maria Pedrera-Canal

**Affiliations:** 1 Metabolic Bone Diseases Research Group, University of Extremadura, 10003 Caceres, Spain; rronmar@unex.es (R.R.-M.); i.aliaga@pdi.ucm.es (I.A.V.); jmlavado@unex.es (J.M.L.-G.); jpedrera@unex.es (J.D.P.-Z.); mariapedreracanal@gmail.com (M.P.-C.); 2 Departamento de Enfermería y Fisioterapia, Universidad de Cádiz, 11009 Cádiz, Spain; luis.moreno@uca.es

**Keywords:** bone density, diet records, dual-energy X-ray absorptiometry scan, peripheral quantitative computed tomography, cross-sectional studies, female, olive oil

## Abstract

Olive oil has been demonstrated to enhance various cardiometabolic risk factors. However, to our knowledge, the association between olive oil intake and cortical and trabecular bone microarchitecture has never been evaluated in Spanish women. We aimed to examine the association between olive oil intake and cortical and trabecular bone microarchitecture. We analyzed 523 women aged 50 (9) year, range (23–81) year. Participants underwent dual-energy X-ray absorptiometry and peripheral quantitative computed tomography scans. Dietary intake of calcium, vitamin D, energy and olive oil (g/day) were assessed by a self-administered food frequency questionnaire (FFQ). After adjustment for potential confounding factors (calcium (mg/day), vitamin D (μg/day) energy (Kcal/day), age, body mass index (BMI) (kg/m^2^), menopausal status, and osteoporotic diagnosis (normal, osteopenia, or osteoporosis)), there were significant increases in volumetric bone mineral density (vBMD) (mg/cm^3^) (*p* < 0.01) in the group with a higher intake of olive oil. Total, trabecular and cortical bone density were positively correlated with olive oil intake. The dietary intake of olive oil was significantly associated with vBMD in multiple regression analysis; total density: olive oil intake (g/day) standardized *β* = 0.185 (*p* < 0.001), trabecular density: olive oil intake (g/day) standardized *β* = 0.186 (*p* < 0.001) and cortical density olive oil intake (g/day) standardized *β* = 0.114 (*p* = 0.008). We conclude that the dietary intake of olive oil is positively associated with a better vBMD in Spanish women.

## 1. Introduction

Olive oil is at the core of the Mediterranean diet and is the major origin of fat within this diet. Olive oil has been recognized for its nutritional quality for centuries [1,2,3]. Dietary components can optimize bone mass and stimulate bone formation [4]. Thus, the intake of olive oil has been related to the prevention of osteoporosis in experimental and in in vitro models [5]. After a follow-up of two years, men aged 55–80 years consuming a Mediterranean diet enriched with virgin olive oil increased robustly their total osteocalcin concentration in parallel to an increased in procollagen I N-terminal propeptide (markers of bone formation), suggesting the protective effects of olive oil on bone [5]. In another clinical follow-up of patients who regularly consumed olive oil over a 1-year period, bone mineral density (BMD) at lumbar vertebrae L3, L4, and the femoral neck had also improved [6]. The relation of bone health and olive oil consumption has also been evaluated by means of calcaneal quantitative ultrasound (QUS), showing that higher T-scores were positively associated with a higher consumption of extra-virgin olive oil [7]. A high consumption of fish and olive oil and low red meat intake has also been positively related to bone mass in adult women, suggesting the potential bone-preserving properties of this dietary pattern throughout adult life [8]. Similarly, a higher adherence to a Mediterranean-like diet is associated with a lower risk of future hip fracture [9]. 

Dietary intake of olive oil has been recommended as an important source of the phenolic compounds that play a role in the prevention of chronic disease and the consequent improvement in quality of life [10]. Those phenolic compounds involve simple phenols (e.g., hydroxytyrosol, tyrosol, caffeic acid, vanillic acid, p-coumaric acid, ferulic acid, and vanillin), flavonoids (e.g., luteolin and apigenin), and others, such as those derived from oleuropein, ligstroside, ligustaloside, verbascoside, and lignans that can positively affect bone health [11,12]. Experimental research and a reduced number of human studies have reported that olive oil, probably thorough its phenolic compounds, may favor bone density maintenance [11], by a mechanism that involve increased bone formation, inhibition of bone resorption and probably decreasing both oxidative stress and inflammation [13].

Currently, the most widely explored approach in the study of olive oil dietary intake and bone health is the study of changes in the fracture rate or the BMD, which are limited by the 2-dimensional analysis of a 3-dimensional structure [14]. Contrary peripheral quantitative computed tomography (pQCT) gives 3-dimensional data of the matrix, providing volumetric density determination, assessment of bone morphology and a separate evaluation of trabecular and cortical bone [14].

There is limited evidence about how olive oil dietary intake might be associated with the bone microarchitecture measured by pQCT. In the present study, we aimed to characterize associations between dietary intake of olive oil and pQCT-derived radial bone characteristics in adult Spanish women.

## 2. Materials and Methods

### 2.1. Study Population

This cross-sectional study includes a convenience sample of participants from the Fish Intake and Bone Health Study (FIBHS), performed in Caceres in Spain. FIBHS was initiated in 2010 with the recruitment of 1865 women (median age 53; interquartile range (IQR) 47–61; range 20–79) from the local area through internet advertising and primary care consults. The study population and selection procedure have been described in detail previously [15]. All the women were within the same ethnic group (Caucasian origin). For the present study, the study sample was contacted in 2017, and a second densitometric study was offered to measure bone volume and bone geometric parameters, including the cortical (outer shell) and trabecular (inner spongy bone) compartments of the forearm. From the initial 1865 components members of the study, 523 (28%) participated. The University of Extremadura Ethical Advisory Committee approved this study. All participants provided written informed consent in accordance with the 1975 Declaration of Helsinki. We aimed to have enough statistical power to detect medium-low effect sizes (anticipated Cohen’s *d* = 0.25) with a *β* = 0.80 and *α* = 0.05, which required a minimum sample size of 506 participants. A total of 523 women were included in this study. In the initial sample, none of the participants were using anti-osteoporotic drugs. In the current subsample, 5 women were undergoing common anti-osteoporotic treatment (1% of the total sample). All subjects led active lives, but none of the women practiced any professional sports. We assessed the participants’ physical activity status on the basis of their answer to the following question: “How much do you exercise or strain yourself physically in your leisure time?” The response categories were (1) sedentary (reading, watching television), (2) moderate (walking, cycling, and exercising in other ways for at least 4 hour per week), (3) active (fitness-improving sport at least three times per week), and (4) competitive sport [16]. Alcohol intake was sporadic and did not exceed 100 mL/day in any case. In total, 75.3% of the participants were non-smokers (*n* = 394).

### 2.2. Anthropometry and Densitometric Study

Height was measured using a Harpenden stadiometer with a mandible plane parallel to the floor, and weight was measured using a biomedical precision balance. Height was measured to the nearest cm and weight to the nearest 100 g. Both measurements were determined when the participants were wearing only light clothing and no shoes. Body mass index (BMI) was calculated as the weight in kilograms divided by the square of the height in meters (kg/m^2^).

Participants underwent a dual-energy X-ray absorptiometry scan (DXA) of their lumbar spine (L2–L4) and hip (left femoral neck) using a Norland XR-800 scanner (Norland at Swissray, Fort Atkinson, WI, USA), and measurements were expressed as the quantity of mineral divided by the area scanned (g/cm^2^). The peripheral quantitative computed tomography (pQCT) measurements were performed on the non-dominant distal forearm using a Stratec XCT-2000 device (Stratec Medizintechnik, Pforzheim, Germany). The scanner was disposed on the distal end of the non-dominant forearm and a coronal computed radiograph (scout view) was implemented. Measurement site was 4% of the forearm length. The XCT-2000 generates 90 projections/image with a slice thickness of 2.0 mm. Voxel size was set at 0.59 mm. The XCT-2000 data were analyzed using version 5.50 of the manufacturer’s software. The pQCT assessment provides a measure of volumetric bone mineral density (vBMD) and distinguishes trabecular from cortical bone. The coefficient of variation (CV %) was below the 2% for all the bone measurements. A pQCT anthropomorphic phantom was scanned at every session to maintain quality assurance. 

### 2.3. Dietary Assessment

Women enrolled in this study completed a 131-item food frequency questionnaire (FFQ). This FFQ was previously validated and involves 24-h recall performed over seven days [15,16,17]. Using the FFQ, we assessed the dietary intake of calcium and vitamin D from the Spanish Food Composition database [18]. The FFQ quantified olive oil consumption in tablespoons per day (one tablespoon of olive oil equals 13.5 g). For some of the calculations performed in the present study, the participants were grouped according to their consumption of olive oil, either above or below/equal to the median intake of the sample (18.32 g/day).

### 2.4. Statistical Analysis

Some of the studied variables were not normally distributed; thus, a two-step approach was used to normalize the data before statistical analyses were conducted, when appropriate [15,19]. This approach included transforming the variable into a percentile rank, followed by applying an inverse normal transformation to the results derived from the first step. Descriptive analyses were conducted for all variables, including mean (SD). The following analyses examined the transformed versions of those variables. Pearson’s correlations were used to explore the relationships between the measured vBMD parameters and the dietary intake of olive oil (g/day). A partial correlation analysis was used to determine the relationships between vBMD and dietary intake of olive oil while controlling for calcium (mg/day), vitamin D (μg/day), energy (Kcal/day), age, BMI (kg/m^2^), menopausal status and osteoporotic diagnosis (normal, osteopenia or osteoporosis). A multiple linear regression (using the stepwise method) was used to examine whether the studied variables (age, BMI (kg/m^2^), calcium intake, vitamin D intake, energy intake and olive oil intake (g/day)) were predictive of selected vBMD parameters. Some subgroup analyses were performed, and comparisons were performed between groups using the unpaired Student’s T-test or the chi-squared test when appropriate. Mean BMD values were compared between groups using an analysis of covariance (ANCOVA), controlling for calcium (mg/day), vitamin D (μg/day) energy (Kcal/day), age, BMI (kg/m^2^), menopausal status and osteoporotic diagnosis (normal, osteopenia or osteoporosis). For the parameter estimations, after further adjustment, the data were reported as the mean (SE). A *p* value of <0.05 was considered statistically significant. All statistical analyses were conducted using IBM SPSS statistical analysis software package (version 22.0) (SPSS Inc., Chicago, IL, USA).

## 3. Results

A total of 523 women participated in the study. The main characteristics of the participants are shown in Table 1 in terms of anthropometric values, nutrient intake, and dietary habits. For quantitative variables, data shown are the mean (SD). Based on the olive oil intake, there were significant differences between groups in the lumbar spine BMD (*p* < 0.01 for all studied areas) and in the vBMD (*p* < 0.01 for all the studied parameters). There were no statistically significant differences in the areal BMD. 

Table 2 describes the associations between dietary olive oil intake (g/day) and the lumbar spine BMD and the vBMD, after further adjustment for calcium (mg/day), vitamin D (μg/day) energy (Kcal/day), age, BMI (kg/m^2^), menopausal status and osteoporotic diagnosis (normal, osteopenia or osteoporosis). The previously reported statistically significant differences in L2, L3, L4, and lumbar spine (L2–L4) remained no longer significant (*p* > 0.05). There were still statistically significant differences between the two studied groups after further adjustment in the total density (*p* < 0.001), trabecular density (*p* < 0.001) and cortical density (*p* = 0.004).

We further explored the main determinants of vBMD by multiple regression analysis. Total density was inversely associated with age (*p* < 0.001) and positively associated with olive oil intake (g/day) and BMI (kg/m^2^) (*p* = 0.006). Trabecular density was positively associated with olive oil intake (g/day) (*p* < 0.001) and BMI (kg/m^2^) (*p* = 0.007), while the cortical density was inversely associated with age (*p* < 0.001) and positively associated with olive oil intake (g/day) (*p* = 0.008) (Table 3).

Finally, there was a significant positive partial correlation between the olive oil intake (g/day) and the vBMD in the studied women. After adjusting for potential confounders (calcium (mg/day), vitamin D (μg/day) energy (Kcal/day), age, BMI (kg/m^2^), menopausal status, and osteoporotic diagnosis (normal, osteopenia, or osteoporosis)), a positive correlation was observed between the total density (*r* = 0.194: *p* < 0.001), trabecular density (*r* = 0.221; *p* < 0.001)and cortical density (*r* = 0.125; *p* = 0.005), and olive oil intake (g/day).

## 4. Discussion

The present study aimed to evaluate the association of olive oil intake and bone density parameters measured by DXA and pQCT. To our knowledge, the present study is the first to explore potential associations between dietary olive oil intake and vBMD. Olive oil is one of the major determinants of the Mediterranean diet, together with fish intake. We have recently reported that the dietary intake of long-chain omega-3 polyunsaturated fatty acid is positively associated with BMD at both the hip and the lumbar spine [15] as well as fish consumption [20]. Taken together, those results are consistent with studies that reflect that a dietary pattern compatible with the Mediterranean diet has positive effects over bone health parameters [7,8,21]. Thus, those results are consistent with the hypothesis that dietary factors may contribute to better bone health. 

After adjusting for important determinants of bone characteristics (calcium (mg/day), vitamin D (μg/day) energy (Kcal/day), age, BMI (kg/m^2^), menopausal status, and osteoporotic diagnosis (normal, osteopenia, or osteoporosis)), none of the differences observed in the BMD (at the lumbar level) in the studied samples remained statistically significant. However, after adjustment, some of the associations initially detected and derived from the pQCT scans remained statistically significant. The different results observed in this study and other previous results reported in relation to BMD and olive oil intake are probably due, in part, to the strict control of potential confounders that we have applied in our study. Previous studies that investigated the effects of olive oil consumption and BMD controlled for a few confounders but not for the whole group of variables that we have used in our analysis. Under such circumstances, it is probable that more weak associations might arise. 

In the present study, we found that women with a higher dietary intake of olive oil (>18.32 g/day) had significantly higher total, trabecular and cortical bone density compared with those with a lower intake of olive oil (≤18.32 g/day). Dietary factors have been reported to prevent cortical bone age decline but without affecting the trabecular bone in elderly women [22]. Particularly, and related to olive oil intake, there is limited literature about the positive association between olive oil consumption or exposure to olive oil components and vBMD. The association of olive oil and bone microarchitecture has been studied in vivo. Our observations are consistent with results that have shown how olive oil supplementation prevents menopause-induced osteoporosis in an ovariectomized rat model [23], improving the cortical and the trabecular bone thickness. In a model of glucocorticoid-induced osteoporosis in the albino rat, olive oil increased the cortical bone thickness [24]. Extra virgin olive oil also qualitatively improved the structure of trabecular and cortical bone mineralization while preserving the size and structure of the mineral crystals in a model of male Wistar rats [25].

Additionally, hydroxytyrosol and oleuropein exposure elevated the femoral trabecular BMD but had no effects on the cortical BMD in a murine model. The efficacy of hydroxytyrosol was higher compared to oleuropein, and this was hypothesized to be due to a higher absorption of hydroxytyrosol vs oleuropein in vivo [26]. However, such results differ from those reported by Tagliaferry and colleagues, who did not observe an improvement of the bone microarchitecture in ovariectomized mice [27] treated with oral olive oil. Other components present in olive oil, such as the flavonoid luteolin, also caused a significant increase in the bone mineral density and bone mineral content of trabecular and cortical bones in the femur compared to those of ovariectomized controls and prevented decreases of bone strength indexes induced by ovariectomy surgery [11,28]. Phytochemicals, antioxidants, and other bioactive compounds have been proposed to have an effect on trabecular bone volume, number, and thickness, and lower trabecular separation by enhancing bone formation and suppressing bone resorption [29]. 

In comparison to DXA, pQCT provides a more refined characterization of bone, including measures of vBMD and bone geometry, and quantifies the distribution of mineral within a cross-section, thus providing a better understanding of the skeletal deficits associated with fracture risk [30]. However, several study limitations must be acknowledged. All of the participants were recruited in our region and surrounding areas, thus different patterns of olive oil consumption across Spain might not be represented. The cross-sectional design does not allow us to establish causal associations between bone density parameters and the dietary intake of olive oil. Olive oil consumption in our study was evaluated by means of an FFQ, which may be unreliable and inadequate for assessing absolute and relative nutrient intakes. The women were enlisted in a convenience sample, which might likely limit the generalizability of our results due to the existence of bias in participant recruitment.

## 5. Conclusions

In conclusion, dietary intake of olive oil is positively associated with total, trabecular and cortical BMD. We encourage further investigations, particularly longitudinal or intervention studies that consider the observed association.

## Figures and Tables

**Table 1:** Biological, anthropometric, dietetic, and densitometric factors in the studied sample by olive oil intake.

	Total Sample	Low (≤18.32 g/day)	High (>18.32 g/day)	*p*-Value
	(*n* = 523)	(*n* = 294)	(*n* = 229)	
Age (years)	50 (9) (23–81)	50 (8) (23–74)	50 (8) (26–81)	0.34
Menarche age (years)	13 (1) (8–19)	13 (1) (8–19)	13 (1)(10–16)	0.897
Gravidity	2 (1) (0–8)	2 (1) (0–8)	2 (1) (0–7)	0.737
Births	2 (1) (0–7)	2 (1) (0–7)	2 (1) (0–6)	0.709
BMI (kg/m^2^)	26.4 (3.8) (19.45–39.60)	26.7 (3.6) (19.45–34.81)	26.1 (3.9) (19.95–39.60)	0.078
Gonadal status				
Premenopausal	284 (54.3%)	163 (55.4%)	121 (52.8%)	0.553
Postmenopausal	239 (45.7%)	131 (44.6%)	108 (47.2%)	
Smoking				
No	394 (75.3%)	216 (73.5%)	178 (77.7%)	0.262
Yes	129 (24.7%)	78 (26.5%)	51 (22.3%)	
Physical Activity				
Sedentary	163 (31.4%)	96 (32.7%)	67 (29.8%)	0.584
Moderate	118 (22.7%)	69 (23.5%)	49 (21.8%)	
Active	238 (45.9%)	129 (43.9)	109 (48.4%)	
Ca (mg/day)	1053.7 (471.7) (175–2834)	961.6 (494.4) (175–2648)	1171.9 (412.6) (244–2834)	<0.001
Vitamin D (μg/day)	7.2 (5.7) (0.34–54.30)	6.5 (5.7) (0.34–54.30)	8.1 (5.6) (0.40–37.88)	0.001
Kcal/day	2181.5 (27.9) (650–4044.7)	2051.7 (583.9) (650–4006.5)	2348.1 (528.2) (992.1–4044.7)	<0.001
Proteins (g/day)	90.1 (30.5) (28.9–227.7)	83.4 (28.7) (31.3–227.7)	98.7 (30.7) (28.9–196.5)	<0.001
Carbohydrates (g/day)	277.7 (91.6) (65.7–639.3)	269.8 (93.7) (65.7–618.6)	287.8 (88.0) (77.8–639.3)	0.026
Fats (g/day)	78.2 (27.9) (27.0–229.9)	71.2 (26.0) (27.0–229.9)	87.2 (27.7) (27.3–223.2)	<0.001
Olive oil (g/day)	20.8 (5.5) (1.83–54.9)	14.8 (2.3) (1.83–18.32)	28.1 (7.4) (20.15–54.96)	N/A
Areal bone mineral density (BMD) (g/cm^2^)				
BMD femoral neck	0.866 (0.098)	0.869 (0.091)	0.861 (0.861)	0.334
BMD femoral trochanter	0.674 (0.087)	0.676 (0.082)	0.671 (0.671)	0.473
BMD Ward’s triangle	0.648 (0.105)	0.651 (0.100)	0.644 (0.644)	0.428
BMD L2	1.055 (0.115)	1.067 (0.106)	1.037 (1.037)	0.002
BMD L3	1.074 (0.116)	1.088 (0.105)	1.055 (1.055)	0.001
BMD L4	1.037 (0.118)	1.053 (0.112)	1.015 (1.015)	<0.001
BMD lumbar spine	1.054 (0.108)	1.068 (0.099)	1.035 (1.035)	0.001
Volumetric BMD (mg/cm^3^)				
Total density	334.278 (37.255)	328.488 (35.006)	341.711 (38.784)	<0.001
Trabecular density	173.171 (27.803)	169.453 (24.642)	177.943 (30.801)	0.003
Cortical density	465.730 (56.283)	459.234 (54.790)	474.068 (57.188)	<0.001
Bone morphometry (mm^2^)				
Total area	303.886 (39.810)	305.823 (39.437)	301.400 (40.233)	0.297
Trabecular area	136.870 (17.901)	137.592 (17.888)	135.943 (17.914)	0.09
Cortical area	166.993 (21.166)	168.376 (20.947)	165.217 (21.360)	0.208
Osteoporotic diagnosis				
Normal	451 (86.2%)	271 (92.2%)	180 (78.6%)	<0.001
Osteopenia	67 (12.8%)	23 (7.8%)	44 (19.2%)	
Osteoporosis	5 (1%)	0 (0%)	5 (2.2%)	

BMI: Body mass index.

**Table 2:** Lumbar BMD and volumetric bone mineral density (vBMD)after further adjustment by potential confounders *.

	Low (≤18.32 g/day)	High (>18.32 g/day)	*p*-Value
	(*n* = 294)	(*n* = 229)	
Adjusted BMD (g/cm^2^)			
BMD L2	1.058 (0.006)	1.051 (0.007)	0.413
BMD L3	1.078 (0.006)	1.069 (0.007)	0.893
BMD L4	1.043 (0.006)	1.029 (0.007)	0.152
BMD lumbar spine	1.059 (0.005)	1.049 (0.006)	0.228
Adjusted volumetric BMD (mg/cm^3^)			
Total density	327.912 (2.165)	342.414 (2.468)	<0.001
Trabecular density	168.203 (1.631)	179.523 (1.859)	<0.001
Cortical density	459.164 (3.288)	474.117 (3.748)	0.004

* Calcium (mg/day), vitamin D (μg/day) energy (Kcal/day), age, BMI (kg/m^2^), menopausal status and osteoporotic diagnosis (normal, osteopenia or osteoporosis).

**Table 3:** Multiple linear regression analysis for the association between vBMD and age, BMI (kg/m^2^), calcium intake, vitamin D intake, energy intake, and olive oil intake (g/day).

**Total Density (mg/cm^3^)**			
Optimal model	*R* ^2^	Adjusted *R*^2^	
	0.065	0.06	
Selected independent variable	Standardized *β*	*t*	*p*-value
Olive oil intake (g/day)	0.185	4.312	<0.001
Age (years)	−0.206	−4.509	<0.001
BMI (kg/m^2^)	0.128	2.781	0.006
**Trabecular Density (mg/cm^3^)**			
Optimal model	*R* ^2^	Adjusted *R*^2^	
	0.043	0.039	
Selected independent variable	Standardized *β*	*t*	*p*-value
Olive oil intake (g/day)	0.186	−4.291	<0.001
BMI (kg/m^2^)	0.118	2.724	0.007
**Cortical Density (mg/cm^3^)**			
Optimal model	*R* ^2^	Adjusted *R*^2^	
	0.051	0.047	
Selected independent variable	Standardized *β*	*t*	*p*-value
Age (years)	−0.198	−4.636	<0.001
Olive oil intake (g/day)	0.114	2.673	0.008
